# Can Neutrophil/Lymphocyte Ratio Assess Inflammatory Bowel Disease Activity and Severity in Children?

**DOI:** 10.5152/tjg.2022.21889

**Published:** 2022-12-01

**Authors:** Rasha Hassan Metwally

**Affiliations:** 1 Department of Pediatrics, Alexandria University Faculty of Medicine, Alexandria, Egypt

**Keywords:** Activity, children, inflammatory bowel disease, severity

## Abstract

**Background:**

Laboratory markers such as white blood cells, C reactive protein, and erythrocyte sedimentation rate can aid in assessing the activity of inflammatory bowel disease but lacks sensitivity and specificity. Fecal calprotectin has higher sensitivity and specificity but it is expensive. Endoscopy is an invasive, inconvenient procedure having complications. No studies are done concerning the neutrophil/lymphocyte ratio in inflammatory bowel disease in pediatrics. The aim of this study was to assess the neutrophil/lymphocyte ratio as a laboratory marker of inflammatory bowel disease activity and severity in children.

**Methods:**

This is a prospective study. The study included all patients from 2 months up to 16 years who were confirmed to have inflammatory bowel disease endoscopically and histopathologically. Clinical activity score and Mayo endoscopic subscore were recorded. Laboratory investigations including white blood cells, C reactive protein, erythrocyte sedimentation rate, and fecal calprotectin were done on all patients. The neutrophil/lymphocyte ratio was calculated and correlated with different activity markers.

**Results:**

We included 50 inflammatory bowel disease patients. The mean neutrophil/lymphocyte ratio in ulcerative colitis was 1.76 ± 0.36, Crohn’s disease was 1.50 ± 0.41, and it was 1.47 ± 0.14 in indeterminate colitis. Neutrophil/lymphocyte ratio was significantly correlated to erythrocyte sedimentation rate, C reactive protein, fecal calprotectin, clinical activity score, and Mayo endoscopic subscore.

**Conclusion:**

Neutrophil/lymphocyte ratio can be used as an activity and severity marker in children with inflammatory bowel disease.

**Main Points**

- Neutrophil/lymphocyte ratio can be used as a marker of activity and severity in inflammatory bowel disease (IBD) patients.
- Endoscopy is an invasive, inconvenient, and expensive method for re-assessing patients with IBD.
- Fecal calprotectin is expensive and needs proper collection and processing of the stool sample.

## Introduction

Inflammatory bowel disease (IBD) is a multifactorial disease characterized by chronic inflammation in the gastrointestinal tract of a genetically predisposed person with periods of remission and exacerbation. The spectrum of IBD in children primarily includes ulcerative colitis (UC), Crohn’s disease (CD), and indeterminate colitis (IC).^1^ The incidence of pediatric IBD is rapidly increasing.^2^

Biomarkers in CD can help in diagnosing and monitoring the activity of the disease. Although endoscopy is the gold standard method for diagnosis and monitoring disease activity of CD, it is an invasive and inconvenient procedure and it may be risky in the setting of severe disease. Both mucosal and clinical evaluations are important in UC. Endoscopy may reveal active mucosal inflammation in the absence of clinical manifestations, and in the same way, clinical remission is not essentially linked to mucosal cure.^3,4^

Disease activity in IBD can be assessed using laboratory markers such as erythrocyte sedimentation rate (ESR) and C-reactive protein (CRP) with sensitivities and specificities ranging between 50% and 60%. Other laboratory markers such as fecal calprotectin and lactoferrin are more specific and sensitive. However, both of them are expensive.^5^ The need for a simple, low-cost marker for disease activity and severity in children is rising.

Systemic inflammatory diseases cause changes in the levels of white blood cells (WBCs). It is agreed that systemic inflammation induces an increase in the number of circulating neutrophils.^6^ The neutrophil/lymphocyte ratio (NLR) is a new biomarker used to diagnose UC and predict the overall disease course.^7,8^ No studies evaluated NLR as a marker for the disease activity and severity in children.

The aim of the present study was to assess the NLR as a marker for IBD severity and activity in children. Moreover, the correlation of the NLR to different activity and severity indices was investigated.

## Materials and Methods

This study was conducted as a prospective study and included all patients from 2 months age up to 16 years who were confirmed to have IBD endoscopically and histopathologically. Formal informed written consent was obtained from the parents or the caregivers of all children before inclusion in the study. The study was conducted in accordance with the ethical standards of Declaration of Helsinki. The study protocol was approved by the ­ethical committee of the Alexandria University Faculty of Medicine, Alexandria, Egypt (ZU-IRB#: 00012098).

The colonoscopic examination along with biopsies was performed on all children included in the study. Gross macroscopic findings were recorded including the severity. The severity of colitis was scored according to the Mayo endoscopic subscore.^9^ All the biopsy specimens were fixed in neutral buffered formalin and they were processed and stained with hematoxylin and eosin stain. Histopathological findings were reported. Classification of the type of IBD (UC, CD, and IC) was done based on both endoscopic and histopathologic findings.

Patients were assessed using pediatric UC activity index (PUCAI) in patients with UC or abbreviated pediatric CD activity index (PCDAI) in patients with CD. The active phase of the disease is defined when PUCAI or the abbreviated PCDAI was 10 or more.^10,11^

The following laboratory investigations were done on all children including complete blood count, CRP, ESR, and fecal calprotectin. Complete blood count with WBC differential was analyzed using a Coulter counter (T660; Beckman Coulter, Brea, Calif, USA). Each type of leukocyte was expressed as a percent of total number of WBCs. The percent value was multiplied by total leukocytic count to calculate the absolute value. The NLR was calculated from differential count by dividing the absolute neutrophil count by the absolute lymphocyte count. Stool sample was obtained prior to administration of laxatives used to prepare patients for colonoscopy and was collected using a disposable plastic bucket-type device to avoid contact with the toilet water and simplify laboratory sampling. In children with diapers, stools were collected directly from the bottom into a test tube to avoid water absorption by diapers which may increase fecal calprotectin value.^12^ Samples were stored at −20°C and thawed at room temperature before testing. Fecal calprotectin was analyzed by a commercially available enzyme-linked immunosorbent assay (Calprest®, Eurospital Spa, Trieste, Italy) with the manufacturer’s recommended cut-off values.^13^

Neutrophil/lymphocyte ratio was correlated to ESR, CRP, fecal calprotectin, clinical activity score, and Mayo endoscopic subscore. Mayo endoscopic subscore was correlated to CRP and ESR. Clinical activity score was correlated to ESR, CRP, fecal calprotectin, and Mayo endoscopic score.

### Statistical Analysis

Data were fed to the computer using Statistical Package for the Social Sciences software package version 20.0 (IBM Corp.; Armonk, NY, USA). Quantitative data were expressed as the mean ± standard deviation, median, minimum, and maximum. For normally distributed quantitative variables, F test (analysis of variance) was used to compare between more than 2 groups and the post hoc test (Tukey) for pairwise comparisons. Pearson coefficient was used to correlate between quantitative variables. The level of significance of .05 was used, equal/below which the results considered to be statistically significant.^14^

## Results

The study included 50 patients. Ulcerative colitis, CD, and IC represented 61.9%, 33.3%, and 4.7%, respectively, of the IBD group. Clinical activity score was calculated and revealed an active disease in all patients; 50% of the patients had mild colitis according to Mayo endoscopic subscore, 9.5% had moderate colitis, and 40.5% had severe colitis.

Mean fecal calprotectin level was 1230.6 ± 868.0 µg/g, mean ESR was 33.90 ± 17.62, mean CRP level was 48.96 ± 39.17, and mean NLR in UC was 1.76 ± 0.36, CD was 1.50 ± 0.41, and it was 1.47 ± 0.14 in IC (Table 1).

Neutrophil/lymphocyte ratio was significantly correlated to ESR, CRP, fecal calprotectin, clinical activity score, and Mayo endoscopic subscore (Table 2). Clinical activity score was correlated to ESR, CRP, fecal calprotectin, and Mayo endoscopic score (Table 3). Mayo endoscopic subscore was significantly correlated to CRP and ESR (*r* = 0.375*P* = .014; *r* = 0.339*P* = .028, respectively)

## Discussion

To our knowledge, WBC, CRP, and ESR are the most commonly used inflammatory laboratory markers in clinical practice for IBD patients. These markers can change with the degree of the inflammation of IBD. Moreover, they lack sensitivity and specificity. On the other side, fecal calprotectin is the best clinically available marker for UC activity with a sensitivity of 93% and specificity of 96%, but it is expensive and needs proper collection and processing of the stool sample.^15^

Multiple studies have assessed the efficacy of circulating leukocyte subtypes as markers of inflammatory disorders including IBD. They established that both absolute neutrophil and lymphocyte counts and their ratios are significantly correlated to the activity in UC.^7,16,17^ In addition, complete blood pictures with differential counts are routinely ordered at the first clinic visit of IBD patients and are hence available.^18^ But data about its use in children are lacking in the literature.

Our study reported that NLR correlates well with other activity and severity indices highlighting the importance of its use as non-invasive low-cost marker of disease severity and activity. This is in line with other studies.^18-20^

Studies conducted on adults reported 2.59 ± 1.47 as a mean level of NLR ratio in active UC, Demir et al^19^ reported 2.39 as a cut-off value for active UC in adults. Feng et al^4^ reported 2.72 cut-off level of NLR in CD in adults. In contrast, we reported lower mean levels of NLR ratio. This may be due to the proportion of lymphocytes and absolute lymphocyte count in children aged younger than 6 years being higher than those in adults.^21^ And so, we recommend that further studies are needed to determine NLR ratio in different pediatric age groups.

Our study is so far an early work on the NLR in IBD patients in children. Moreover, some clinical scores such as PUCAI or abbreviated CDAI are not suitable for IC highlighting the importance of the presence of another activity and severity marker. The cut-off value that indicates active colitis needs to be studied. More large-scale studies are needed to study NLR in IBD in different pediatric age groups.

## Conclusion

From this study, it can be concluded that NLR can be used as an activity and severity marker in children with IBD.

## Figures and Tables

**Table 1.** Comparison Between the 3 Studied Groups According to Different Parameters

	**IBD Group**			**Test of Significance**	***P***
	**Ulcerative Colitis (n =30)**	**Crohn’s Disease (n = 18)**	**Indeterminate Colitis (n = 2)**		
**Neutrophils (%)**					
Min.–Max.	50.0-66.0	28.0-65.0	55.0-55.0	*F *= 3.961^*^	.027^*^
Mean ± SD	59.46 ± 5.40	53.21 ± 9.07	55.0 ± 0.0		
Median	60.0	53.50	55.0		
**Significance between groups**	*P*_1_ = .022^*^, *P*_2_ = .646,* P*_3_ = .936				
**Lymphocytes (%)**					
Min.–Max.	30.0-45.0	30.0-57.0	35.0-40.0	*F *= 0.928	.404
Mean ± SD	34.65 ± 4.85	36.93 ± 6.55	37.50 ± 3.54		
Median	35.0	35.0	37.50		
**Neutrophils/lymphocytes ratio**					
Min.–Max.	1.11-2.17	0.4-2.17	1.38-1.57	*F *= 2.434	.101
Mean ± SD	1.76 ± 0.36	1.50 ± 0.41	1.47 ± 0.14		
Median	1.71	1.53	1.47		

^*^Statistically significant at *P* ≤ .05.

F, analysis of variance test, a pairwise comparison between each 2 groups was done using post hoc test (Tukey); *P*, comparison between the studied groups; *P*_1_,comparison between ulcerative colitis and Crohn’s disease; *P*_2_, comparison between ulcerative colitis and indeterminate colitis; *P*_3_, comparison between Crohn’s disease and indeterminate colitis;min, minimum; max, maximum; SD, standard deviation.

**Table 2.** Correlation Between Neutrophils/Lymphocytes Ratio with Different Studied Parameters in IBD Cases (n = 50).

**IBD Cases (n = 50)**	**Neutrophils/Lymphocytes Ratio**	
	**R**	***P***
**Clinical activity score **	0.322^*^	.037^*^
ESR	0.398^*^	.009^*^
Fecal calprotectin	0.318^*^	.040^*^
CRP	0.313^*^	.043^*^
Endoscopic Mayo score	0.159	.314

^*^Statistically significant at *P* ≤ .05.

ESR, erythrocyte sedimentation rate; CRP, C reactive protein; IBD, inflammatory bowel disease; *r*, Pearson coefficient.

**Table 3.** Correlation Between Clinical Activity Score with Different Studied Parameters in IBD Cases (n = 50)

**IBD Cases (n = 50)**	**Clinical Activity Score**	
	**R**	***P***
ESR	0.355^*^	.021^*^
Fecal calprotectin	0.328^*^	.034^*^
CRP	0.658^*^	<.001^*^
Endoscopic Mayo score	0.320^*^	.039^*^

^*^Statistically significant at *P* ≤ .05.

ESR, erythrocyte sedimentation rate; CRP, C reactive protein; IBD, inflammatory bowel disease; *r*, Pearson coefficient.
